# Emerging Themes from EBV and KSHV microRNA Targets

**DOI:** 10.3390/v4091687

**Published:** 2012-09-21

**Authors:** Dhivya Ramalingam, Philippe Kieffer-Kwon, Joseph M. Ziegelbauer

**Affiliations:** HIV and AIDS Malignancy Branch, National Cancer Institute, National Institutes of Health, Bethesda, MD 20892, USA; Email: dhivya.ramalingam@nih.gov (D.R.); philippe.kieffer-kwon@nih.gov (P.K.-K.)

**Keywords:** EBV, KSHV, HHV4, HHV8, miRNAs, microRNAs

## Abstract

EBV and KSHV are both gamma-herpesviruses which express multiple viral microRNAs. Various methods have been used to investigate the functions of these microRNAs, largely through identification of microRNA target genes. Surprisingly, these related viruses do not share significant sequence homology in their microRNAs. A number of reports have described functions of EBV and KSHV microRNA targets, however only three experimentally validated target genes have been shown to be targeted by microRNAs from both viruses. More sensitive methods to identify microRNA targets have predicted approximately 60% of host targets could be shared by EBV and KSHV microRNAs, but by targeting different sequences in the host targets. In this review, we explore the similarities of microRNA functions and targets of these related viruses.

## 1. Introduction

Approximately twenty percent of human cancers are associated with various infectious agents [1]. Two such agents are Epstein-Barr virus (EBV/human herpesvirus 4) and Kaposi’s sarcoma-associated herpesvirus (KSHV/human herpesvirus 8). Other viruses directly linked to human cancers include hepatitis B virus (HBV), hepatitis C virus (HCV), human papillomavirus (HPV), human T-cell lymphotropic virus (HTLV-1), and Merkel cell polyomavirus. EBV and KSHV are members of the herpesvirus family that can infect lymphocytes and are associated with various proliferative disorders. EBV associated disorders include Burkitt’s lymphoma (BL), Hodgkin’s lymphoma (HL), NK/T-cell lymphoma, diffuse large B-cell lymphoma (DLBCL) post-transplant lymphoproliferative disorder, and nasopharyngeal carcinoma (NPC) (reviewed in [2]). KSHV infections are associated with Kaposi’s sarcoma, primary effusion lymphoma (PEL) and multicentric Castleman’s disease (MCD). Both gamma-herpesviruses, EBV and KSHV have evolved to maintain life-long latent infections in their human hosts. Both viruses can infect B-lymphocytes, while in vivo evidence shows that EBV can also infect epithelial cells and KSHV also infects endothelial cells. 

The complex steps involved in viral oncogenesis have largely been characterized by studying the interactions between human and viral proteins. A new class of molecules- microRNAs, have begun to be appreciated in virus-host interactions starting with the discovery of virally-encoded microRNAs in EBV [3] and in other herpesviruses, including KSHV [4]. It is believed that EBV and KSHV microRNAs are processed in the same fashion as human microRNAs (though viral microRNA processing can follow a unique pathway in some viruses [4,5]). MicroRNAs have been reviewed extensively elsewhere [6], but briefly, they are short (~22 nt) single-stranded RNA molecules that arise as RNA polymerase II primary transcripts after a number of processing steps. Once the mature microRNAs are generated, they can be incorporated into the RNA-induced silencing complex (RISC), where they can target mRNAs through imperfect complementarity to suppress gene expression. Repression of target mRNAs can trigger destabilization of the mRNA followed by degradation and/or inhibit protein translation. In the context of latent viral infection, these are attractive molecules to modulate host gene expression since they require little viral genome space, avoid generation of additional potentially antigenic viral latent proteins, and are unlikely to be selectively targeted by the host, since humans may lack the ability to distinguish the viral miRNA sequences from human miRNA sequences. Viruses may utilize miRNAs to exploit a faster evolution rate given that a change of few nucleotides can dramatically change the repertoire of targeted mRNAs. Finally, recent studies with mutant strains of viruses, which lack certain miRNAs imply that viral miRNAs play important roles in maintaining the latent/lytic switch [7,8].

Multiple insightful reviews have covered the cellular and viral targets of viral miRNAs [9,10,11,12,13]. Here we focus on the cellular targets of EBV and KSHV miRNAs and look for similarities in the targets and functions of miRNAs from these related viruses. As a result of multiple deep-sequencing efforts, we currently feel we have discovered all of the EBV and KSHV microRNAs. Although the miRNAs from both these viruses lack sequence similarity, one similarity is in their genomic organization. Both viruses show clustering of the miRNA genes and most miRNAs come from the same starting primary miRNA transcript. In KSHV, the 12 microRNA genes are largely in one cluster in the latency locus while the 25 microRNAs of EBV, are largely in two clusters- BART miRNAs and three BHRF1 miRNAs [14].

A variety of approaches have been employed to identify the targets of miRNAs. Bioinformatic methods can be used to find targets through identification of mRNA sequences that are complementary to the seed region of miRNAs (reviewed in [15]). Frequently, miRNA targets are identified by scanning the 3’ untranslated regions (UTRs) of mRNAs for sequences complementarity. Additionally, gene expression studies can be used to find gene products that are altered when viral miRNAs are introduced or inhibited [16,17,18]. Recent methods utilize microarrays or the sensitivity of deep sequencing to find mRNA sequences that are biochemically purified with the RNA-induced silencing complex (RISC) [19,20,21,22]. In crosslinking and immunoprecipitation (CLIP) assay, miRNAs and their targets are UV crosslinked to the RISC proteins. The ternary Ago-miRNA-mRNA complexes are then purified by immunoprecipitation of the Ago protein. Immunoprecipitated mRNAs are converted to cDNA and analyzed by high throughput sequencing (HITS-CLIP). HITS-CLIP generates a genome wide-map of miRNA binding sites, but the direct position of interaction between mRNA and protein is not precisely identified. To enhance the poor crosslink efficiency between RNAs and proteins provided by UV irradiation, neo-synthesized RNAs could be labeled with photoactivatable ribonucleoside enhanced crosslinking and immunoprecipitation (PAR-CLIP) such as 4-thiouridine (4-SU). Furthermore, crosslinked 4-SU induces a thymidine to cytidine transition during the process and consequently marks the position of successfully crosslinked sequences. Comparing CLIP reads between non-infected and infected cells allows the determination of specific targets of viral miRNAs. However, to identify the precise miRNA binding sites a bio-informatic analysis based on seed sequence homology is often required. Each method has inherent advantages and limitations, but the combination of these approaches have been successful in identifying many miRNA targets (Table 1). After identifying miRNA targets using bioinformatic or cell culture-based screening methods, a variety of assays are used to validate predicted miRNA targets. The most common way to validate a miRNA binding site is to clone this binding site in the 3’UTR of a luciferase reporter and to express this reporter in the presence of the miRNA. An effective binding of the miRNA to a target mRNA then represses luciferase activity of the reporter. These assays are essentially identical to validation studies performed with cellular miRNAs, with the advantage that the uninfected cells are ideal control cells for introducing viral miRNAs. Additionally, the numbers of different viral miRNAs expressed in an infected cell is experimentally more manageable than the hundreds of cellular miRNAs expressed in a given cell type. 

Here, we explore the similarities and differences in the functions of miRNA targets from the limited number of experimentally validated miRNA targets and the larger number of targets predicted by recent reports using high-throughput sequencing of RNA isolated by crosslinking immunoprecipitation (HITS-CLIP and PAR-CLIP) [20,21,22] (Table 2). The exact significance of these targets needs to be further investigated to understand their potential roles in infection and viral-host interactions. Nevertheless, some main cellular functions emerged from the miRNA targets of EBV and KSHV miRNAs.

**Table 1:** List of experimentally validated cellular targets of KSHV- and EBV- encoded miRNAs. The normal cellular functions of these proteins and the effect of their knockdown during viral infection are also listed. The targets that are underlined are repressed by both KSHV- and EBV-encoded miRNAs. ND- No data.

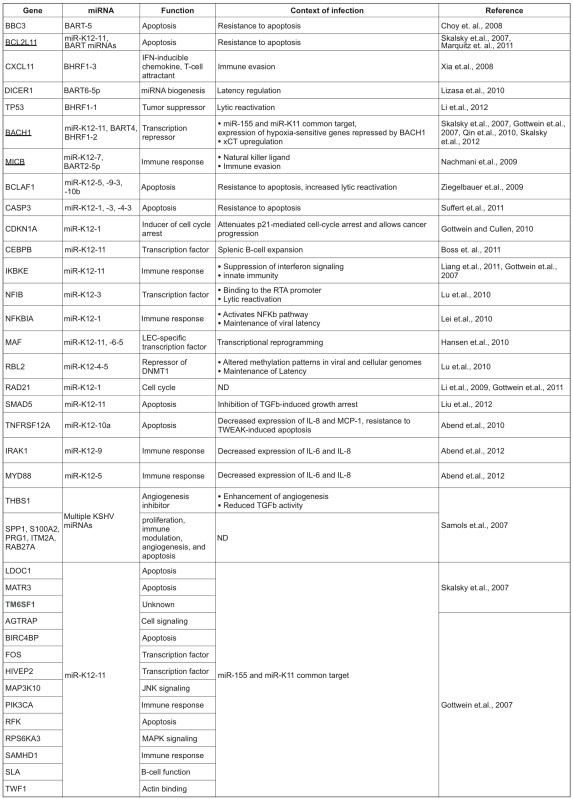

**Table 2:** Validated targets from high-throughput studies. Shown are cellular targets identified by photoactivatable ribonucleoside enhanced crosslinking and immunoprecipitation (PAR-CLIP), High-throughput sequencing of RNA isolated by crosslinking immunoprecipitation (HITS-CLIP) or RNA immunoprecipitation and microarray (RIP-Chip) techniques. Validation of these targets was done using luciferase reporter assays.

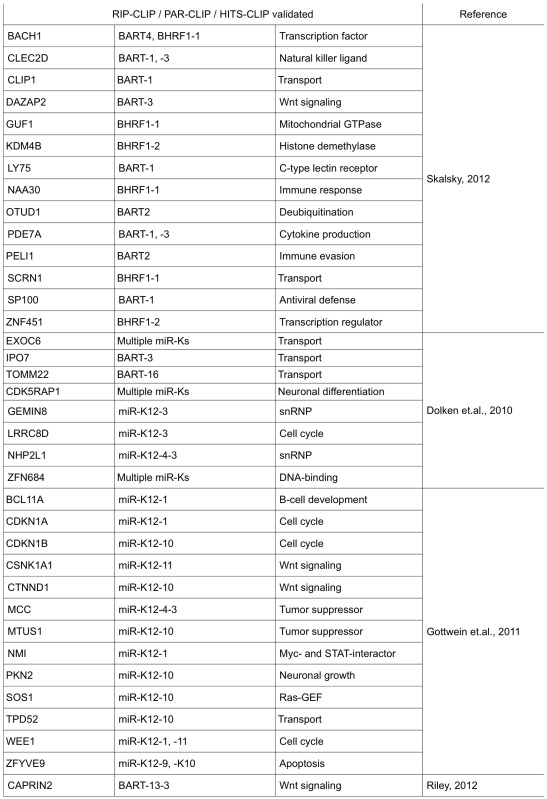

## 2. Immune evasion strategies by viral miRNAs

Although 90% of adults throughout the world are infected by EBV, most of the infected people are healthy and do not develop EBV-related carcinoma. In vivo, the immune system restrains EBV-infected cells to latency I and II. EBV- infected cells that undergo latency III program generate a strong and specific T- cell response. However, the latently infected cells persist in the host and can, in rare cases, cause cancers such as Burkitt’s Lymphoma (BL) from infected B cells or nasopharyngeal carcinoma (NPC) from infected epithelial cells. Several proteins of γ-herpesviruses were described to thwart immune defense (reviewed in [23]). Recently, viral microRNAs of γ-herpesviruses have been shown to play a significant role in immune evasion of infected cells. 

EBV takes advantage of its microRNAs to disturb the communication between infected B-cells and other cells involved in the immune response. The first identified target of an EBV miRNA implicated in immune-evasion was the IFN-inducible T-cell attracting chemokine, CXCL-11/I-TAC [24]. B-cells activated by IFN-γ produce CXCL-11, a ligand for the CXCR3 receptor on NK-cells and Th1 lymphocytes. Inhibition of BHRF1-3 in BL enhances CXCL-11 secretion whereas over-expression of BHRF1-3 in those cells decreases CXCL-11 induction by IFN-γ. 

The major histocompatibility complex class I–related chain B (MICB) is a stress-induced protein recognized by the NKG2D receptor on NK-cells and CD8+ T-lymphocytes. Interaction between MICB and NKG2D generates a cytolytic response by NK- and T-cells. Nachmani et.al. demonstrated that the 3’UTR of MICB is targeted by EBV miRNA, BART2-5p and by the KSHV miRNA, miR-K12-7. In both cases, viral miRNAs decreased expression of MICB on the cell surface and consequently reduced cell death mediated by activation of NKG2D [25]. This is one of the rare host genes that have been found to be targeted by both KSHV and EBV miRNAs, but this class of co-targeted host genes is likely to expand as more targets are discovered. 

In addition, CLEC2D/LLT1 was identified as a target of BART1 and BART3 by PAR-CLIP [21]. CLEC2D is expressed on the surface of B-cells after viral infection or upon activation of toll-like receptor (TLR) or B-cell receptor signaling pathways [26]. CLEC2D is recognized by CD161 on NK- and T- cells and induces production of IFN-γ by CD8+ T lymphocytes. Although it has not been shown that inhibition of CLEC2D decreases IFN-γ production, direct targeting of BART1 and BART3 in the 3’UTR of CLEC2D was demonstrated by luciferase assays. Paradoxically, an up-regulation of CLEC2D by EBV B95-8 on peripheral B cells was also observed [26]. Skalsky et al. [21] used well-established latency III Lymphoblastoid Cell Lines (LCL) whereas Germain et al. studied de novo infected peripheral blood mononuclear cells (PBMCs) 66 hours after infection. Those different experimental conditions might lead to different expression patterns of EBV genome and therefore may explain difference in CLEC2D expression.

Other target genes of EBV miRNAs identified by PAR-CLIP and associated with immune evasion are: LY75/CD205, PDE7A, PELI1 and SP100. For each of them, direct interactions between 3’UTR and EBV miRNAs were confirmed by luciferase assay but no functional assay has been performed so far (Table 2). In dendritic cells, transmembrane receptor LY75 (lymphocyte antigen 75) carries antigens from surface of the cell to late endosomes or lysosomes. Consequently, LY75 mediates antigen presentation to CD8+ and CD4+ T-cells via MHC-I and –II receptors [27]. Knockdown of LY75 by BART1 and BHFR1 should help LCL cells avoid recognition from CD4+/CD8+ T-cells. Cyclic nucleotide phosphodiesterases (PDE) hydrolyse cAMP and thus modulate second messengers of numerous pathways. PDE7A is also required for cytokine production of peripheral T cells [28] and PDE7A is involved in proliferation of NKT cells [29]. Surprisingly PDE7A-/- mice do not have any defect in their T-cell lineage [30]. BART1 and BART3-3p target the 3’UTR of PDE7A and the exact benefits to the EBV life cycle are currently unknown. PELI1 is an E3 ubiquitin ligase activated by IRAK1 or MyD88, downstream of IL-1R or TLR pathway respectively. Ubiquitination of various substrates by PELI1 (e.g. RIP1, TRAF) leads to NF-κB activation and induces expression of pro-inflammatory cytokines and chemokines [31]. Knockdown of PELI1 by BART2-5p might decrease the inflammatory response from EBV-infected cells. Another strategy used by EBV to establish infection is to disturb the intrinsic anti-viral defense of the host. SP100 is a key component of promyelocytic leukemia-nuclear bodies (PML-NB/NB10). Although, the precise antiviral function of PML-NB is not fully understood, γ-herpesviruses have developed multiple strategies to disassemble these nuclear bodies (reviewed in [32]). Knockdown of SP100 by BART1-5p may be one such method developed by EBV to inactivate PML-NB. 

In addition to MICB, several genes involved in immune response are down-regulated by the miRNAs of KSHV. Recently, components downstream of TLR and IL-1 signaling pathways, IRAK1 and MYD88, have been shown to be repressed by KSHV miRNAs and inhibit IL-6 and IL-8 secretion from IL-1-stimulated cells [81]. Furthermore, IKBKE/IKKε is a non-canonical I-kappa-B kinase down-regulated by miR-K12-11. One substrate of IKBKE is the interferon regulatory factor 3 (IRF3), downstream of the TLR signaling pathway. Therefore, repression of IKBKE by miR-K12-11 attenuates IFN signaling and decreases the antiviral response of the host [33]. Interestingly, the NF-kappa-B inhibitor NFKBIA/IκBα is also down-regulated by KSHV-miR-K12-1, leading to NFKB activation and maintenance of latency (see below) [33]. Identified by PAR-CLIP, RFXAP is a direct target of miR-K12-1 in a PEL cell line [20]. Previous data has shown that RFXAP is an essential MHC-II regulatory gene [34]. Regulation of MHCII genes is essential to establish an immune response, but the significance of RFXAP down-regulation in the context of KSHV infection remains unknown. 

Interestingly, it was reported that the immunomodulatory protein galectin 9 and the EBV miRNAs could be transferred from NPC or LCL cells to non-infected cells via exosomes [35,36]. Pegtel et al. demonstrated that miRNAs present in the exosomes are functional and able to down-regulate their targets in neighboring cells. They showed that exosomes carrying BHRF1-3 can down-regulate CXCL11 in dendritic cells. Consequently, the immuno-suppression effect of viral miRNAs should be not only considered on infected cells but also on adjacent cells. Such transfer of viral miRNAs has not been reported for KSHV so far. However, if the transfer of miRNAs via exosomes is a common feature among γ-herpesviruses, newer studies addressing this field will likely result in important discoveries.

## 3. Regulation of apoptosis by KSHV and EBV miRNAs

Resistance to apoptosis is a common mechanism evolved by many viruses to persist in the host and many cellular targets of KSHV and EBV miRNAs are involved in apoptotic pathways. Using a microarray-based approach to identify mRNAs whose levels were repressed in the presence of KSHV miRNAs, BCLAF1 (Bcl-2-associated factor), a pro-apoptotic protein, was identified as a target of miR-K12-5, -9, -3 and -10b. The repression of BCLAF1 by KSHV miRNAs enabled the cells to overcome etoposide-induced caspase activation [18]. Virally encoded miRNAs can also attenuate cellular responses to extracellular signals as demonstrated in the case of TNFRSF12A (TWEAKR), which is the cellular receptor for the TNF-like weak inducer of apoptosis (TWEAK). Knockdown of TWEAKR levels by miR-K12-10a protected cells from TWEAK-induced apoptosis and also reduced the levels of the cytokines- IL-8 and MCP-1, secreted in response to TWEAK-induction [37]. In another study, caspase 3 was itself identified as a target of multiple KSHV miRNAs, which conferred resistance to apoptosis [38]. In addition, many proteins involved in apoptosis regulation like the tumor suppressors- p53 (for EBV- BHRF1-1) [39], LDOC1 (leucine zipper down-regulated in cancer 1; KSHV miR-K12-11) [40] and MTUS1 (microtubule associated tumor suppressor 1) [20] have been validated. KSHV miR-K12-1 down-regulated the levels of the cyclin-dependent kinase inhibitor, p21, thereby allowing the infected cells to overcome p21-mediated cell cycle arrest [41]. Further, HITS-CLIP and PAR-CLIP studies have identified numerous proteins involved in Wnt signaling (CAPRIN2, CTNND1, DAZAP2, CSNK1A1) as targets of KSHV and EBV-encoded miRNAs [20,21,22]. 

The pro-apoptotic proteins, BIRC4BP/XAF1 (XIAP associated factor), PUMA (p53-upregulated modulator of apoptosis) and BCL2L11 (or Bim) were also down-regulated by KSHV or EBV miRNAs [17,40,42]. PUMA and Bim are BH3-only proteins of the Bcl-2 family that inhibit the anti-apoptotic functions of Bcl-2 and knockdown of these proteins by EBV-BART-miRNAs conferred cell survival [42,43,44]. Further, both PUMA and Bim are activated by two transcription factors- p53 and C/EBPβ, that are themselves regulated by viral/ virally-induced miRNAs (see below). Interestingly, Bim was repressed by both KSHV and EBV miRNAs, demonstrating the significance of Bim knockdown in the biology of these viruses [40,44]. 

The TGF-β pathway confers a strong anti-proliferative phenotype to many epithelial and endothelial cells and hence, it is presumable that KSHV has developed mechanisms to escape from this inhibition. SMAD5, the downstream effector of TGF-β is repressed by KSHV- miR-K12-11, and this allowed the cells to overcome the cytostatic effects mediated by TGF-β and enhanced cell proliferation [45]. In addition, thrombospondin 1 (THBS1) (KSHV; see below) and DAZAP2 (EBV; BART3) are proteins of the TGF-β pathway that are repressed by viral miRNAs [16,21]. The TGF-β pathway is also down-regulated via a different mechanism by KSHV. The LANA protein was shown to inhibit TGFβ-type II receptor expression by epigenetic silencing in PEL cells [46]. This is an example of synergy between latently expressed miRNAs and proteins to modulate the same cellular pathway. This redundancy will likely be observed with other miRNA targets as viruses routinely use redundant mechanisms. An additional method of promoting growth is by EBV modulating expression of the human miRNA, miR-34a [82]. 

## 4. Control of lytic reactivation by viral miRNAs

Controlling the latency to lytic transition is a critical step in the life cycle of herpesviruses. While latency appears to be the default pathway for most herpesviruses during which they can evade host immune responses, they also need to be able to proceed into the lytic stage when the conditions are favorable. KSHV and EBV have also evolved miRNAs that target both cellular and viral proteins to control this transition in a temporal manner. Deletion of a cluster of miRNAs in the KSHV genome (except miR-K12-10 and -12), results in enhanced RTA expression and virion production. As RTA is an activator of lytic genes and triggers virus reactivation, this suggests that the miRNAs inhibited lytic reactivation. IκBα, the inhibitor of NFκB, was identified as the cellular target repressed by KSHV miR-K1. IκBα knockdown resulted in an increased activation of NFκB and facilitated maintenance of latency [8]. Yet another component of the NFκB pathway that is repressed by KSHV miRNAs is IKKε. miR-K12-11-mediated down-regulation of IKKε attenuated interferon signaling and inhibited IKKε -mediated KSHV reactivation [33]. Thus, by activating a cellular survival pathway via NFκB, KSHV is able to maintain latency, suppress antiviral responses and enhance proliferation of the latently infected cells. 

As with KSHV, EBV uses its miRNAs to control the switch between latent and lytic infection [83]. Dicer was shown to be a target of EBV-BART6 and the regulation of dicer levels by this miRNA controlled the stage of infection (latency-I, II, III or lytic). Reduction in dicer levels by BART6 resulted in a suppression of genes that facilitate lytic replication like ZTA/RTA, whereas inhibition of BART6 via antagomirs reversed this phenotype [47]. With KSHV, repression of BCLAF1 by miR-K12-5 also promoted lytic reactivation [18], while p53 was repressed by EBV- BHRF1-1 during lytic reactivation [39]. KSHV-miR-K12-3 could repress the levels of the transcription factor, nuclear factor I/B (NFIB). The promoter of RTA was shown to contain an NFIB binding site and hence, NFIB could enhance RTA expression and lytic replication. Thus, by repressing the levels of NFIB, KSHV can maintain latency [48]. Deletion of a cluster of KSHV miRNAs (miRs-K12-1-9 and -11) resulted in altered patterns in histone modifications and loss of DNA CpG methylation. Further analysis revealed that miR-K12-4-5 repressed the levels of Rbl2, an inhibitor of cellular DNA methyltransferases (DNMTs) and regulator of the cell cycle. miR-K12-4-5-mediated knockdown of Rbl2 resulted in the activation of DNMTs, thereby silencing several viral and cellular genes [7]. Thus KSHV miRNAs also contribute towards maintaining the latency-lytic switch via epigenetic mechanisms.

In addition to these cellular mRNAs, many viral mRNAs including RTA of KSHV and BALF5, the EBV DNA polymerase, are also directly repressed by viral miRNAs to prevent lytic reactivation. RTA controls the latency-lytic switch of KSHV and was repressed by multiple KSHV miRNAs- miR-K12-9-5, -7-5 and -5 [7,49,50]. BART2 is expressed in the antisense direction from the BALF5 3’-UTR and hence, has complete sequence complementarity to BALF5 mRNA. Repression of BALF5 by BART2 was reduced during lytic reactivation and so, BART2 might facilitate the maintenance of EBV latency [51].

## 5. Regulation of angiogenesis by viral miRNAs

Angiogenesis, the development of new blood vessels from existing vasculature, is a hallmark of Kaposi’s sarcoma. The virus has evolved mechanisms to overcome cellular inhibitors to this process. One of the first validated KSHV-miRNA targets was THBS1, a potent inhibitor of angiogenesis and proliferation. THBS1 levels were found to be repressed by many KSHV-encoded miRNAs, including, miR-K12-1, -K3-5, -K6-3 and –K11. Reduced THBS1 levels further resulted in reduced TGFβ activity, as demonstrated using luciferase reporter assays [16]. In addition, the authors also identified several genes involved in angiogenesis and proliferation, whose mRNA levels were repressed over 4-fold in microarrays upon expression of KSHV miRNAs. These genes include SPP1 (osteopontin), S100A2 (S100 Calcium binding protein A2), PRG1 (plasticity related gene 1) and ITM2A (integral membrane protein 2A), but the functional significance of the knockdown of these mRNAs in the context of KSHV infection is not clear [16].

## 6. Cellular transport regulation by viral miRNAs

xCT (or SLC7A11) is a member of the heteromeric family of cystine/glutamate antiporters that exchange intracellular glutamate for extracellular cystine; the cystine is rapidly converted to glutathione within the cell and hence, xCT protects cells from oxidative stress [52]. Recently, xCT was also identified as the fusion receptor for KSHV entry [53]. KSHV miRNAs, miR-K12-1, -9 and –11, were shown to upregulate the levels of xCT, by repressing BACH1. High xCT levels increased the susceptibility of the macrophages to KSHV infection and allowed for the infected cells to survive in an environment of high reactive nitrogen species (RNS) [54]. 

Using Ago2-based RIP-CHIP analysis, two cellular transport-related proteins- TOMM22 (mitochondrial import receptor subunit TOMM22 homolog) and IPO7 (importin 7; involved in nuclear import of proteins) were identified and validated as EBV-BART16 and –BART3 targets, respectively, using 3’UTR luciferase assays [19]. EBV can take advantage of the repression of TOMM22 and IPO7 since siRNA-mediated knockdown of TOM22 inhibits BAX-induced apoptosis [55], while siRNAs targeting IPO7 in macrophages resulted in reduced IL-6 levels upon LPS stimulation [56]. In addition, RIP-Chip and PAR-CLIP analysis have revealed proteins of the intracellular vesicle transport and exocytosis machinery- EXOC6 (exocyst complex component 6; multiple KSHV-miRNAs) [19], SCRN1 (secernin 1; EBV-BHRF1-1) [21], TPD52 (tumor protein D52; KSHV-miR-K12-10) [20] and CLIP1 (CAP-GLY domain containing linker protein 1; EBV-BART-1) [21], as targets of KSHV and EBV-encoded miRNAs. 

Dölken et al. have shown that expression of the 10 intronic pri-miRNAs represses a luciferase reporter carrying the 3’UTR of EXO6C [19]. EXO6C is a subunit of the vesicle exocyst-tethering complex and contributes to exocytosis by promoting fusion between secretory vesicles and plasma membrane [57]. Furthermore, in HeLa cells knockdown of EXO6C considerably reduces the final exocytic events [58]. It is difficult to hypothesize what advantage KSHV could get from blocking exocytosis. However, blocking exocytic events might reduce cytokine or chemokine secretion that could disturb proper loading of MHC receptors on the cell surface of infected cells. Further research is required to confirm this hypothesis. 

BART1 binds the 3’UTR of CLIP1/CLIP-170 as demonstrated using PAR-CLIP and luciferase assays [21]. CLIP1 anchors endosomes to the microtubule network and its expression is upregulated in B-cells stimulated with IL-4/CD40L. Consequently, CLIP1 takes part in antigen presentation and its down-regulation by EBV might decrease recognition of infected cells by CD4+/CD8+ T-cells. Lastly, a gene down-regulated by EBV miRNAs that is associated with intracellular traffic is ZNF451. ZNF451 is a nuclear zinc-finger protein without any intrinsic transcription activation function; the 3’UTR of its mRNA is directly targeted by BHRF1-3 [21]. The advantages of ZNF451 repression for EBV is not yet understood. Hence, it is conceivable that EBV and KSHV miRNAs target cellular proteins involved in numerous transport processes to persist in an unfavorable environment, resist apoptosis and overcome the innate immune response of the host.

## 7. Similarities between miR-155 and miR-K12-11 in the context of KSHV/ EBV infection

One interesting discovery in the study of virally encoded miRNAs was the identification of the KSHV miRNA, miR-K12-11, as the viral ortholog of the cellular miRNA, hsa-miR-155 (miR-155) [17,40]. Two independent groups showed that miR-K12-11 and miR-155 have the exact ‘seed’ sequence and therefore, could regulate a similar set of genes. The common set of genes that were down-regulated by both miRNAs included BACH1 (BTB and CNC homology 1), a transcription repressor involved in the regulation of genes regulating cell cycle and oxidative stress [59]. In addition, by microarray profiling and luciferase assays, the authors validated LDOC1 (leucine zipper down-regulated in cancer 1), MATR3 (Matrin 3), TM6SF1 (transmembrane 6 superfamily member 1), PIK3CA (phosphoinositide-3-kinase, catalytic α subunit), XAF1, HIVEP2 (HIV-1 enhancer binding protein 2) and Fos as common targets of both miRNAs [17,40]. 

In order to identify miR-K12-11 as a true functional ortholog of miR-155, Boss et.al. stably expressed either miR-K12-11 or miR-155 in CD34+ human cord blood progenitor cells and studied immune reconstitution using the NOD/LtSz-scid IL2Rγnull mouse model [60]. These mice have neither circulating complement nor functional NK, B, T, APC cells, and they are deficient in cytokine signaling. Therefore, they can be humanized by transplantation of human cord blood derived CD34+ progenitors and use as an *in vivo* model for KSHV infection. It was found that the expression of miR-K12-11 or miR-155 repressed the levels of C/EBPβ, a transcription factor that negatively regulates IL-6 and contributes to the development of many B-cell lymphomas. Further, this repression of C/EBPβ by either miR-K12-11 or miR-155 resulted in an increased proliferation of B-cells in the spleen, contributing to lymphomagenesis [60]. More recently, it was shown that SMAD5 is a direct target of both miR-K12-11 [45] and miR-155 [61]. The repression of SMAD5 by both miRNAs was demonstrated to overcome TGFβ -induced growth arrest, thereby, leading to increased cell division and tumor development [45,61]. 

miR-155 is an oncomiR found to be upregulated in many B-cell lymphomas [62,63]. Interestingly, EBV, which does not encode an ortholog of miR-155, upregulates miR-155 upon infection and this is important for B-cell immortalization [64]. Further, the α-herpesvirus, Marek’s Disease Virus (MDV) expresses MDV-miR-M4, an ortholog of miR-155. The two miRNAs were shown to regulate a common set of genes including C/EBPβ and HIVEP2, that are also validated targets of KSHV-miR-K12-11 [65]. Together, these studies suggest that the viral orthologs of hsa-miR-155 may perform overlapping functions and the deregulation of the target genes of these miRNAs might contribute to the development of B-cell lymphomas. 

## 8. Other targets of KSHV and EBV encoded miRNAs

In addition to repressing factors involved in apoptosis, immune evasion and cell cycle arrest, several proteins involved in splicing, miRNA biogenesis and transcription factors have been identified as targets of KSHV and EBV miRNAs. In RIP-CHIP analysis of KSHV infected cells, two proteins of the small nuclear ribonucleoprotein (snRNP) family- NHP2L1 (non-histone chromosome protein 2-like 1) and GEMIN8 (gem (nuclear organelle) associated protein 8), were repressed by KSHV miRNAs, miR-K12-4-3 and -3, respectively. Interestingly, the binding sites for these two miRNAs were found to be in the coding regions of these two genes. In the same study, the authors also identified the CDK5 inhibitor, CDK5RAP1 (CDK5 regulatory subunit associated protein 1), which is predominantly involved in neuronal differentiation and neuroskeletal differentiation, as a target of KSHV miRNA [19]. As mentioned earlier, Dicer, the key enzyme involved in miRNA biogenesis, was also identified as the target of EBV-BART6-5 [47]. 

In addition, several transcription factors were identified as targets of KSHV miRNAs, among which, ZFN684 [19], BACH1 [17,40], HIVEP2 and Fos [17] have been validated. Transcription factors were among the most enriched family of proteins in PAR-CLIP analyses of both KSHV and EBV infected cells [20]. KSHV infection of lymphatic endothelial cells (LECs) causes transcriptional reprogramming and induces the expression of markers specific for blood vessel endothelial cells (BECs) [66]. Down-regulation of the LEC-specific transcription repressor, MAF, by KSHV miRNAs- miR-K12-6 and miR-K12-11, was shown to induce the expression of several BEC-specific markers like CXCR4, thereby contributing to transcriptional reprogramming [67]. 

## 9. Overlap of targets from KSHV and EBV high-throughput miRNA studies

More recently miRNA targets have been predicted using more sensitive approaches of high-throughput sequencing of RISC-associated mRNAs in KSHV and/or EBV infected cell lines [20,68,69]. These methods yield short mRNA sequences that are associated with RISC and through searching these sequences for seed-matching sequences, an association between a specific mRNA and miRNA can be predicted. All of these reports have shown the ability to use this technology to identify validated miRNA targets using 3’UTR luciferase assays and Western blot assays. Here we elucidate the similarities among the genes predicted to be targets from these studies. However, we share the limitations of this analysis. First, the definition of a predicted target differs across the various reports. Second, the completeness of predicted targets made publicly available differed between the reports. Third, the mRNAs predicted to be viral targets are dependent on the sequencing reads possessing a perfect seed-matching site. This raises the possibility that certain mRNA targets being targeted by viral miRNAs using non-canonical seed-matching are not included in the list of predicted hits. Additionally, if a human miRNA having the same seed-match site as a viral miRNA targets an mRNA, then the mRNA would be erroneously labeled as a target of both miRNAs. This analysis does not distinguish miRNA sequences beyond the seed region. Finally, long 3’UTRs, certain secondary structures, and base composition may influence transient, but non-biologically relevant cross-linking events resulting in a predicted miRNA target site. Nevertheless, it is a worthwhile exercise to investigate the similarities and differences of the predicted targets using an additional filter for finding viral miRNA targets, to reveal similar viral-host interactions, and to possibly identify differences in the virus-host interactions between KSHV and EBV. While the specific target sites are unlikely to be conserved between these viruses, recent data suggests a significant overlap of miRNA targets of these viruses. The specific genes targeted and the classes of targets may improve our understanding of viral oncogenesis and immune evasion.

The overlap of targets of EBV and KSHV miRNAs has been approached using biochemical purification of RISC followed by microarrays to measure mRNAs associated with RISC in EBV and KSHV infected cell lines [70]. This analysis predicted 114 targets for KSHV miRNAs and 44 targets for EBV miRNAs. Analysis of these predicted targets revealed only three genes were predicted as miRNA targets for both KSHV and EBV miRNAs (PDCD1LG2, JARID1B, and SOCS3). These genes have been shown to be involved with immune response [71], protein demethylation [72], and cytokine responses [73]. KSHV miRNA targets showed an enrichment of gene ontology terms for genes involved in splicing, gene expression and protein import into the nucleus [70].

**Figure 1:**
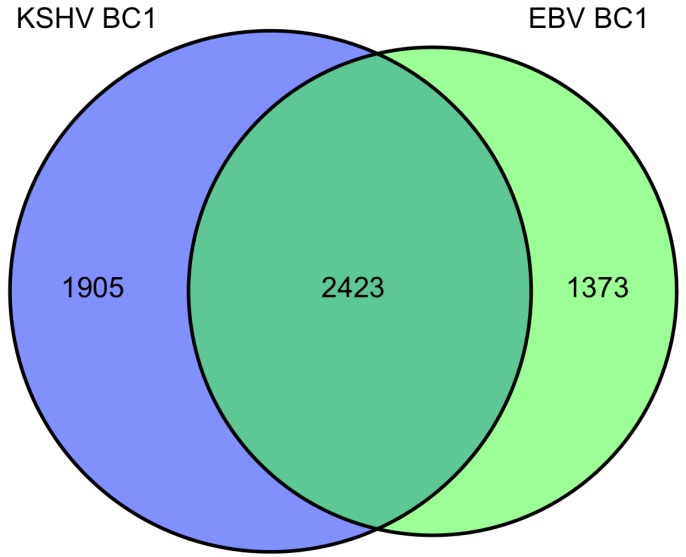
Venn diagram of BC1 predicted miRNA targets from Gottwein et al. 2011.

We investigated the overlapping genes across the various lists of predicted targets from PAR-CLIP [20,68] and HITS-CLIP [69] assays. For the first analysis, we considered the KSHV and EBV miRNAs that are all naturally expressed in the primary effusion cell line, BC-1 (which do not express the BHRF1 EBV miRNAs). In the original report using only 3’UTR predicted targets, it was observed that fifty-eight percent of KSHV targets were also predicted to be targets of EBV miRNAs. In the analysis here, we included all coding sequence (CDS) and 3’UTR predicted targets. Of the KSHV miRNA predicted targets (3796 genes), sixty-four percent were also EBV predicted targets (Figure 1). Likewise, of the EBV miRNA predicted targets (4328 genes), fifty-six percent are also KSHV predicted targets. Initially, this high degree of correlation may seem surprising since there are no KSHV and EBV miRNAs which share the same seed region. However, this suggests KSHV and EBV miRNAs are both targeting a majority of similar genes through different target regions within the same transcripts and these viral miRNAs are playing similar roles in modulating gene expression. Using the 2423 overlapping genes predicted to be targets of EBV and KSHV miRNAs in the co-infected BC-1 cell line, we analyzed this list for enrichment of certain pathways or process networks (manually curated gene sets based on pathways and gene ontology terms; similar to gene set enrichment analysis) in a process similar to measuring enrichment of gene ontology terms (using MetaCore from Thomson Reuters). As shown in Table 3, there was a significant enrichment of pathways involved in cytoskeleton remodeling. Endothelial cells undergo morphological changes to form spindle cells upon KSHV infection [74,75]). Additionally, KSHV utilizes clathrin-mediated endocytosis and actin remodeling an important role in KSHV entry into cells [76]. Another pathway that is enriched in the EBV and KSHV targets is the PTEN pathway. The predicted targets in this pathway include p21 [41], p53, PTEN, caspase-9, FOXO3, and others. A KSHV protein, LANA, has been shown to inhibit p53 [77], suggesting that KSHV latent proteins and miRNAs can cooperate to inhibit p53. FOXO3 was found to be poorly expressed in nasopharyngeal carcinoma tissues and may represent an important marker for this EBV-related cancer [78]. In the enriched process networks in Table 3, the networks related to cell cycle control and apoptosis are expected based on earlier work on p21 [41], caspase 3 [38] and others [18]. Perhaps more surprising, was the network of genes involved in translation initiation. While translation inhibition upon viral infection has been investigated since 1964 [79], it has been generally described during lytic infection (unlike the latent infection in BC-1 cells). Inspection of the overlapping genes revealed twelve genes from the eukaryotic translation initiation family. Together, this analysis reveals some common themes of predicted KSHV and EBV miRNA targets in BC-1 cells. 

**Table 3:** BC1 overlapping miRNA target associations.

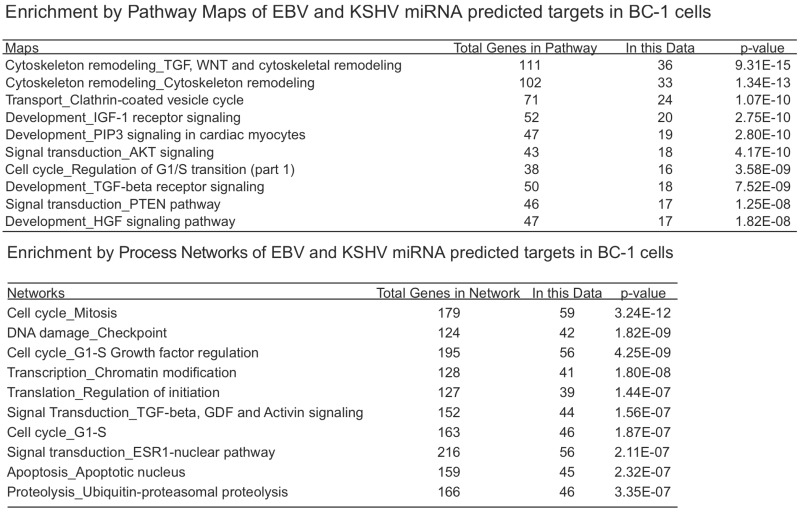

**Figure 2:**
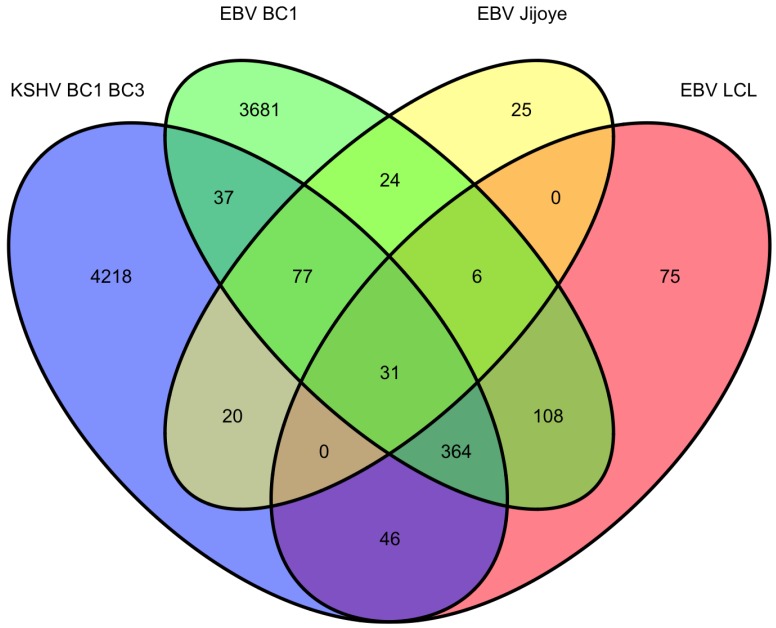
Venn diagram of BC1 predicted miRNA targets from Gottwein et al. 2011.

**Figure 3:**
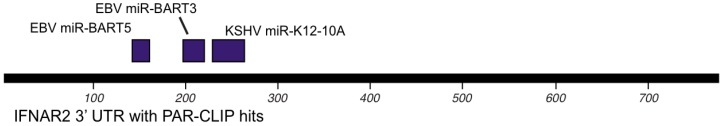
Map of interferon receptor 2 (IFNAR2) 3’UTR with miRNA target sites as determined by CLIP assays.

As mentioned above, there are four datasets from PAR-CLIP or HITS-CLIP assays looking at EBV and KSHV miRNA targets. As shown in Figure 2, there were 31 genes that were found in all four datasets. Given, the differences between the methods used, arbitrary cutoffs, miRNA expression differences among different cell lines, we sought to investigate a broader group of genes. We selected 478 genes (472 are predicted KSHV miRNA targets) that were found in at least three of the four datasets. Using a similar analysis as above, we analyzed this set for functional themes. Again, we observed enrichment of EBV and KSHV predicted miRNAs involved in cell cycle regulation, apoptosis, cytoskeleton remodeling (Table 4). Additionally, we observed a theme of predicted targets involved in immune responses, specifically genes in the B-cell receptor pathway, interleukin regulation, and the interferon pathway. This analysis highlighted interferon receptor 1 and 2 as predicted miRNA targets in all of the datasets, except the EBV Jijoye dataset [22]. But, it should be noted that all of the predicted targets from this dataset were not publicly available, raising the possibility that this was predicted in this dataset as well. Closer analysis shows the PAR-CLIP analysis yielded RISC-associated clusters that are distinct for each viral miRNA, yet in a same region (10% of the 3’UTR sequence length) near the 5’end of the 3’UTR (Figure 3). Could this reflect a region of the mRNA that is less bound by obstructing RNA binding proteins and/or mRNA structures that are more accessible to RISC? Answering these questions could improve bioinformatic methods to identify miRNA targets.

**Table 4:** Three of four overlapping miRNA target associations. Shown are the enriched associations of miRNA targets identified in at least three of the four CLIP datasets shown in Figure 2.

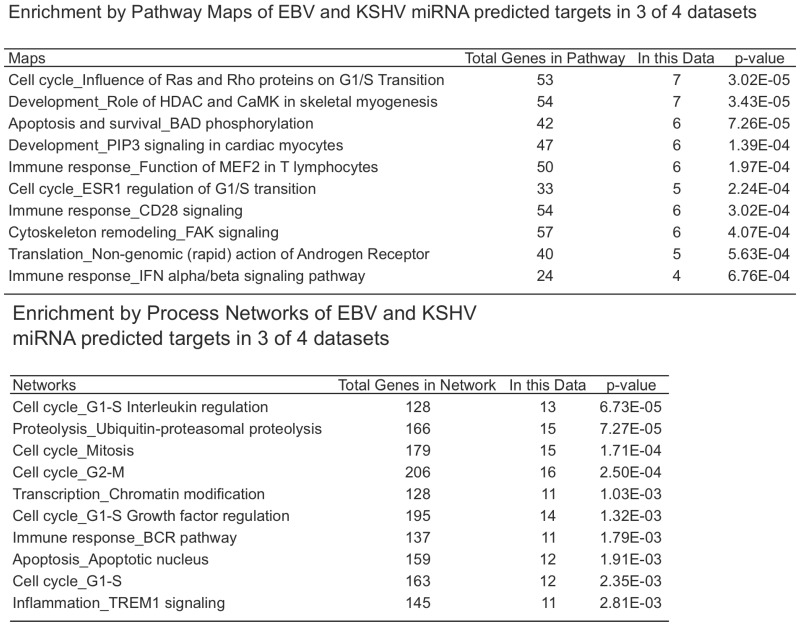

**Figure 4:**
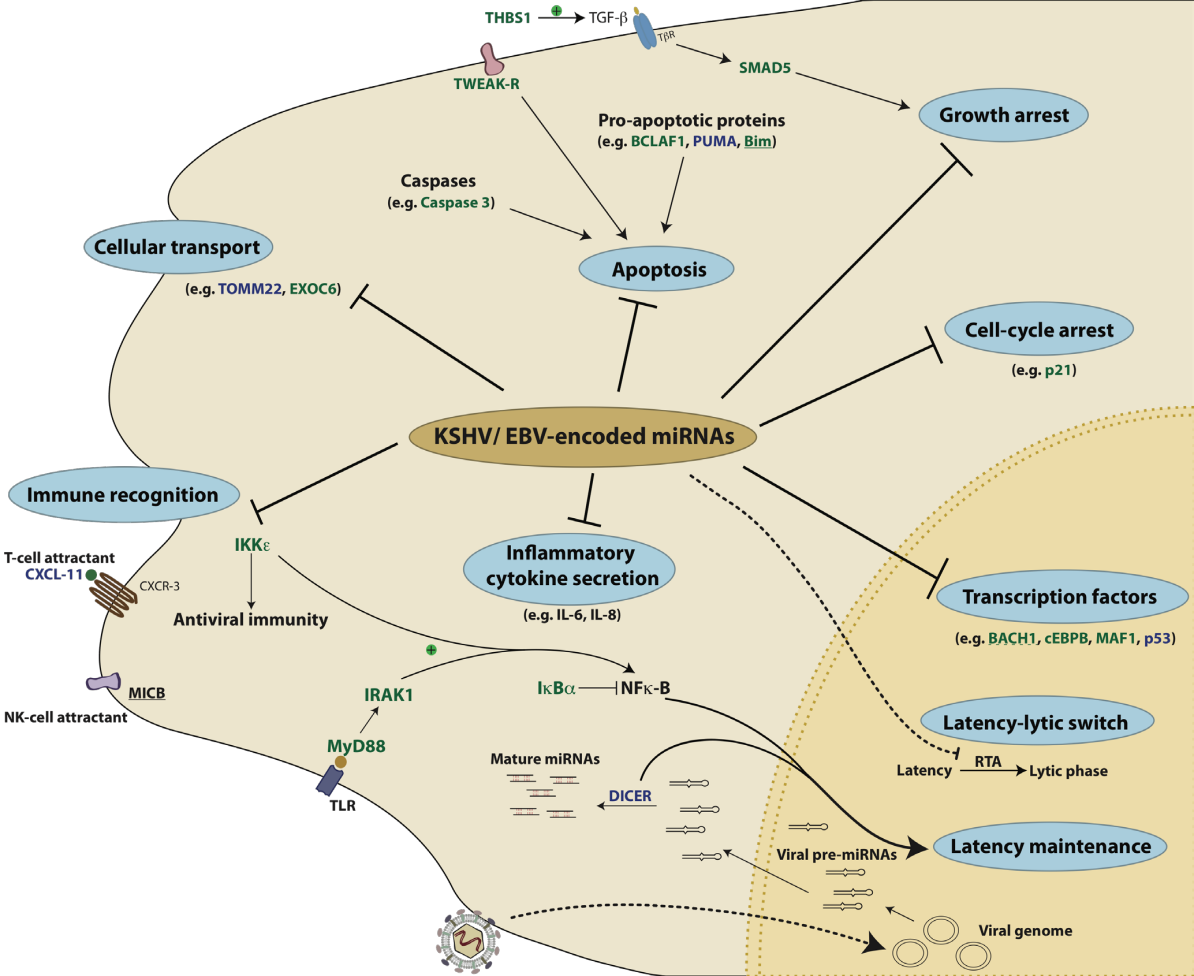
KSHV- and EBV-encoded miRNAs target several key pathways to establish disease. 1) Viral miRNAs can inhibit pro-apoptotic proteins like Bim and BCLAF1. 2) Viral miRNAs can also enable the infected cells to evade recognition by the immune system by repressing either the T-cell attracting chemokine, CXCL-11 or the NK-cell attracting ligand, MICB. 3) miRNAs can also inhibit the production of interferons and inflammatory cytokines by repressing factors like IKKε, IRAK1 and MyD88. 4) By repressing factors like IκBα and Dicer, viral miRNAs can also regulate latency. In addition to cellular targets, the RTA protein of KSHV that acts as the master regulator of the latency-lytic switch, is itself a target of KSHV miRNAs. 5) Several transcription factors like BACH1, c/EBPβ and p53 are repressed by KSHV and EBV miRNAs. 6) KSHV miRNAs repress p21 and SMAD5 to overcome p21- and TGFβ- mediated growth arrest, respectively. In this illustration, the targets of KSHV miRNAs are in green, those of EBV miRNAs are in blue and targets that are repressed by both KSHV and EBV are underlined. The dotted line represents a viral target of KSHV-encoded miRNAs.

## 10. Conclusions

As time progresses, more discoveries will be shared about additional miRNA targets. Additionally, how these targets interact with each other and with viral proteins remains to be further studied. Future research will likely include investigating the roles of exosomes in manipulating the host outside of infected cells. Furthermore, understanding the roles of viral miRNAs during infection in vivo remains to be a challenge for the field and will likely be complicated by redundant functions of miRNAs and possibly viral proteins. One goal for the field is beyond identifying miRNA targets, but using viral miRNAs as tools to show us humans what the virus already knows is important for repression. A deeper understanding of the networks of miRNA targets (Figure 4) will improve our understanding of viral pathogenesis and yield new insights into the functions of host proteins and virus-host interactions.
